# The probability of a sequential *Plasmodium vivax* infection following asymptomatic *Plasmodium falciparum* and *P. vivax* infections in Myanmar, Vietnam, Cambodia, and Laos

**DOI:** 10.1186/s12936-019-3087-1

**Published:** 2019-12-30

**Authors:** Lorenz von Seidlein, Pimnara Peerawaranun, Mavuto Mukaka, Francois H. Nosten, Thuy-Nhien Nguyen, Tran Tinh Hien, Rupam Tripura, Thomas J. Peto, Tiengkham Pongvongsa, Koukeo Phommasone, Mayfong Mayxay, Mallika Imwong, James Watson, Sasithon Pukrittayakamee, Nicholas P. J. Day, Arjen M. Dondorp

**Affiliations:** 1 Mahidol Oxford Tropical Medicine Research Unit, Faculty of Tropical Medicine, Mahidol University, Bangkok, Thailand; 2 Centre for Tropical Medicine and Global Health, Nuffield Department of Medicine, University of Oxford, Oxford, UK; 3 MShoklo Malaria Research Unit, Mae Sot, Thailand; 4 Oxford University Clinical Research Unit, Wellcome Trust Major Oversea Programme, Ho Chi Minh City, Vietnam; 5 Center of Tropical Medicine and Travel Medicine, Department of Infectious Diseases, Amsterdam University Medical Centers, Meibergdreef, University of Amsterdam, Amsterdam, The Netherlands; 6 Savannakhet Provincial Health Department, Savannakhet, Savannakhet Province Lao PDR; 7 Department of Clinical Tropical Medicine, Faculty of Tropical Medicine, Mahidol University, Bangkok, Thailand; 8 Lao-Oxford-Mahosot Hospital-Wellcome Trust Research Unit (LOMWRU), Microbiology Laboratory, Mahosot Hospital, Vientiane, Lao PDR; 9 Amsterdam Institute for Global Health & Development, AHTC, Amsterdam, Netherlands; 10 Institute of Research and Education Development, University of Health Sciences, Vientiane, Lao PDR; 11 Department of Molecular Tropical Medicine and Genetics, Faculty of Tropical Medicine, Mahidol University, Bangkok, Thailand; 12 The Royal Society of Thailand, Dusit, Bangkok, Thailand

**Keywords:** *P. falciparum*, *P. vivax*, Primaquine, Radical cure, Universal

## Abstract

**Background:**

Adding 8-aminoquinoline to the treatment of falciparum, in addition to vivax malaria, in locations where infections with both species are prevalent could prevent vivax reactivation. The potential risk of haemolysis under a universal radical cure policy using 8-aminoquinoline needs to be weighed against the benefit of preventing repeated vivax episodes. Estimating the frequency of sequential *Plasmodium vivax* infections following either falciparum or vivax malaria episodes is needed for such an assessment.

**Methods:**

Quarterly surveillance data collected during a mass drug administration trial in the Greater Mekong Subregion in 2013–17 was used to estimate the probability of asymptomatic sequential infections by the same and different *Plasmodium* species. Asymptomatic *Plasmodium* infections were detected by high-volume ultrasensitive qPCR. Quarterly surveys of asymptomatic *Plasmodium* prevalence were used to estimate the probability of a *P. vivax* infection following *Plasmodium falciparum* and *P. vivax* infections.

**Results:**

16,959 valid sequential paired test results were available for analysis. Of these, 534 (3%) had an initial *P. falciparum* monoinfection, 1169 (7%) a *P. vivax* monoinfection, 217 (1%) had mixed (*P. falciparum *+ *P. vivax*) infections, and 15,039 (89%) had no *Plasmodium* detected in the initial survey. Participants who had no evidence of a *Plasmodium i*nfection had a 4% probability to be found infected with *P. vivax* during the subsequent survey. Following an asymptomatic *P. falciparum* monoinfection participants had a 9% probability of having a subsequent *P. vivax* infection (RR 2.4; 95% CI 1.8 to 3.2). Following an asymptomatic *P. vivax* monoinfection, the participants had a 45% probability of having a subsequent *P. vivax* infection. The radical cure of 12 asymptomatic *P. falciparum* monoinfections would have prevented one subsequent *P. vivax* infection, whereas treatment of 2 *P. vivax* monoinfections may suffice to prevent one *P. vivax* relapse.

**Conclusion:**

Universal radical cure could play a role in the elimination of vivax malaria. The decision whether to implement universal radical cure for *P. falciparum* as well as for *P. vivax* depends on the prevalence of *P. falciparum* and *P. vivax* infections, the prevalence and severity of G6PD deficiency in the population and the feasibility to administer 8-aminoquinoline regimens safely.

*Trial registration* ClinicalTrials.gov Identifier: NCT01872702, first posted June 7th 2013, https://clinicaltrials.gov/ct2/show/NCT01872702. This study was registered with ClinicalTrials.gov under NCT02802813 on 16th June 2016. https://clinicaltrials.gov/ct2/show/NCT02802813

## Background

Novel approaches to the curative treatment and prevention of vivax malaria are urgently needed to achieve the elimination of malaria. Currently reductions in vivax malaria prevalence and incidence are lagging behind the more successful falciparum malaria elimination efforts [1]. Unlike *Plasmodium falciparum*, *Plasmodium vivax* infections relapse weeks to months after the initial attack [2]. Repeated relapses cause considerable morbidity, misery, and loss of income in vivax endemic areas [3]. Relapsing infections are also a persistent source of gametocytes, fuelling *P. vivax* transmission [4]. The triggers for hypnozoite activation are not completely understood, but acute febrile illness and by-products of haemolysis have been proposed [5–7].

The observation that people living in co-endemic regions have an increased rate of vivax malaria following a falciparum malaria episode compared to those who did not have a recent falciparum malaria episode suggests that in co-endemic regions a falciparum infection is a risk factor for vivax relapse [7, 8]. The risk of vivax malaria following falciparum malaria has been estimated as low as zero in several locations and as high as 65% in Papua-New Guinea [9, 10]. The lack of efficacy of the schizontocidal treatment against recurrent vivax infections and the timing of relapses has been interpreted as evidence that vivax recurrences following falciparum malaria are due to reactivation of *P. vivax* hypnozoites [11]. However, the available molecular tools are not able to discriminate whether a *P. vivax* infection is a relapse or a new infection [12]. In co-endemic regions, it has been proposed that “universal radical cure” be given for both *P. vivax* and *P. falciparum* infections [9].

The only class of drugs that can eliminate hypnozoites and hence prevent vivax relapse are the 8-aminoquinoline primaquine and tafenoquine [13, 14]. The small but real risk of haemolysis in glucose-6-phosphate dehydrogenase (G6PD) deficient individuals after the administration of 8-aminoquinoline regimens is a major barrier to the uptake of radical curative regimens and slows the elimination of vivax malaria. With the increasing availability of robust and accurate point of care tests for G6PD deficiency, health care providers are increasingly able to prescribe 8-aminoquinoline to clear vivax infections without putting the patient at risk. There is a broad consensus on the benefits of adding a course of 8-aminoquinoline to the schizontocidal treatment of vivax malaria. Detecting and treating asymptomatic *P. vivax* carriers is more challenging. In co-endemic regions *P. falciparum* infections could serve as a marker for earlier *P. vivax* infections. In such a scenario, the inclusion of 8-aminoquinoline in the treatment *P. falciparum* infections in addition to vivax malaria (universal radical cure), could benefit the *P. vivax* infected patient and accelerate the elimination of *P. vivax*. The relative benefits of such proactive treatment depend to a large part on the probability of an episode of *P. vivax* parasitaemia following a *P. falciparum* infection. To get a better understanding of such potential benefits this study explores the probabilities of sequential *Plasmodium* infections using data from a trial of mass drug administrations (MDAs) in villagers living in four countries of the Greater Mekong Subregion (GMS).

## Methods

The data for the current study were collected during a cluster randomized trial conducted between 2013 and 2017 in Myanmar, Vietnam, Cambodia, and Laos [15]. The aim of the trial was to assess the effectiveness, safety, tolerability, and acceptability of mass administrations of three rounds of dihydroartemisinin–piperaquine (DHA–PPQ) with a single low dose primaquine (SLD PQ). The MDAs were conducted at months 0, 1, 2 in intervention villages. The MDA intervention was allocated by restricted randomization within pairs of villages matched for geographical proximity and parasite prevalence. Of the 4423 people residing during the MDAs in the 8 intervention villages 3790 (86%) completed at least one round (3 doses) of anti-malarials. In addition, there were 294 new-comers registered until month 12. The 4310 residents in 8 control villages at month 0 plus 733 new-comers who joined later were invited to participate in cross-over MDAs after 12 months (M12, M13, M14) with the exception of the residents in two control villagers in Myanmar who were offered MDAs at M9, M10, M11. The surveillance data analysed in the current study are from the first 12 months in the control and intervention villages in Myanmar, Vietnam, Cambodia, and Laos and 9 months in the control villages in Myanmar. The month 12 data from the control arm in Myanmar are not included in the analysis as cross-over MDA took place at month 9 because of accessibility concerns during the rainy season.

### Surveillance

At M0, directly preceding the MDA in intervention villages and subsequently every 3 months, all residents of the study villages aged 6 months or older were invited to participate in cross-sectional prevalence surveys, including temporary inhabitants and migrant workers arriving after the MDA was completed. The presence or absence of each participant in the village during the previous period was assessed during the quarterly surveys. Venous blood (3 mL) was collected from all individuals aged ≥ 5 years, and 500 µL from children aged ≥ 6 months to 5 years. Participants with fever ≥ 37.5 °C were tested for malaria by rapid diagnostic tests (RDT) and malaria positive cases were treated according to national guidelines.

### Laboratory

The blood samples were stored in a cool box in the field and then transported within 12 h to the local laboratory and processed by separation of plasma, buffy coat, and packed red blood cells, which were frozen and stored at − 80 °C. The frozen samples from Myanmar, Cambodia, and Lao PDR were transported monthly on dry ice to the Department of Molecular Tropical Medicine and Genetics in Bangkok, Thailand for DNA extraction, and high-volume ultrasensitive quantitative Polymerase Chain Reaction (uPCR). The samples from the Vietnam sites were shipped to the Oxford University Clinical Research Unit in Ho Chi Minh City, Vietnam for DNA extraction, and uPCR. Detailed description and evaluation of the uPCR methods have been reported previously [16].

### Statistical analysis

The conditional probability of a *P. vivax* infection in a current survey was calculated given the *Plasmodium* infection status 3 months earlier (the previous survey), which could be a *P. falciparum*, a *P. vivax*, a mixed or no infection. Thus, the data point of each participant included in this analysis had the same exposure period. Only the status of infection 3 months earlier was included in this analysis. The risk of *P. vivax* infections following *P. falciparum* or *P. vivax* infections was assessed using risk ratios. The risk ratios were calculated as the ratio of the conditional probabilities of *P. vivax* following *P. falciparum* or *P. vivax* infections in the preceding survey to the conditional probability of having a *P. vivax* infection when there was no *Plasmodium* species detected previously. Since participants could contribute more than one episode of malaria species infection, we used the Generalized Estimating Equation (GEE) model to account for repeated observations in the same study participant. A GEE model with log binomial link function was fitted to the outcome (present of subsequent *P. vivax* infection) conditional on the preceding infection status (*P. falciparum*, *P. vivax*, mixed or no infection). The conditional probabilities, risk ratio and their 95% confidence interval were obtained. The risk difference (RD) was calculated as the difference between the assumed cure rate of primaquine minus the observed conditional probability of having no subsequent *P. vivax* infection when *P. falciparum* was detected at the time of the survey 3 months earlier. The risk differences that account for clustering were calculated from the conditional probabilities. The 95% confidence intervals were calculated by first obtaining the standard error of the difference in probabilities. The standard errors were calculated by squaring each of the standard errors of the probabilities which were then summed up and the square root taken. Then the 95% confidence interval for the risk differences was calculated in the usual way of risk difference plus or minus 1.96 multiplied by the standard error.

Next, the number of *P. falciparum* infected individuals needed to treat (NNT) with 8-aminoquinoline in order to prevent one *P. vivax* infection where NNT = 1/(risk difference) were estimated. The estimates make the assumption that the radical cure using an appropriate dose of primaquine has a 99% cure rate, i.e. nearly all subsequent *P. vivax* infections could have been prevented if the participants been treated appropriately. We also estimated the number of *P. vivax* infected people needed to treat (NNT) with 8-aminoquinoline in order to prevent sequential same species *P. vivax* infections. The 95% confidence intervals for NNT were calculated by obtaining the inverse of the lower and upper limits of the 95% confidence intervals for the risk difference and reversed their order [17]. The standard error of the risk difference was assumed to be the same in the observed data and in the hypothetical data (in which cure rate was assumed to be 99%). The analysis was performed in Stata 15.0.

## Results

Of the 9760 residents living in the 16 villages during the 12-month study period, 6235 residents (1372 from Myanmar, 2004 from Vietnam, 1267 from Cambodia, and 1592 from Laos) contributed 16,959 valid sequential paired test results included in this analysis. Of these, 534 (3%) had a *P. falciparum* monoinfection, 1169 (7%) a *P. vivax* monoinfection, 217 (1%) had mixed (*P. falciparum *+ *P. vivax*) infections, and 15,039 (89%) had no *Plasmodium* infection in the initial survey.

As shown in Table 1, of the 534 participants who had an initial monoinfection with *P. falciparum*, 47 had a subsequent *P. vivax* infection detected at next survey (9%; 95% Confidence Interval: 7% to 12%). Of 1169 participants who had an initial mono *P. vivax* infection, 584 had a subsequent *P. vivax* infection detected at the next survey (45%; 95% CI 42% to 48%). Of the 217 participants with mixed *P. vivax* and *P. falciparum* infection 104 were found to have a subsequent *P. vivax* infection at the next survey (47%; 95% CI 40 to 54%). Out of the 15,039 participants who were initially found to be uninfected 515 had subsequently *P. vivax* infections (4%; 95% CI 3 to 4%).

**Table 1:** Conditional probabilities of subsequent *P. vivax* infections adjusted for correlation among qPCR test result from same individual

Plasmodium parasites detected in the preceding survey	n/N	Probability of a subsequent *P. vivax* infection (95% CI)	RD (95% CI)	RR (95% CI)
*P. falciparum* (monoinfection)	47/534	0.090 (0.068, 0.119)	0.054 (0.029, 0.080)	2.4 (1.8, 3.2)
*P. falciparum *+ *P. vivax* (mixed infection)	104/217	0.466 (0.402, 0.540)	0.430 (0.361, 0.498)	12.3 (10.3, 14.6)
*P. vivax* (monoinfection)	584/1169	0.447 (0.416, 0.480)	0.411 (0.379, 0.442)	12.2 (11.0, 13.6)
Negative	515/15,039	0.036 (0.033, 0.039)	Reference	Reference

*RD* risk difference, *RR* relative risk

The risk of subsequent *P. vivax* infections following *P. falciparum* monoinfections was about two-fold increased (Risk Ratio 2.4, 95% CI 1.8 to 3.2) compared to the risk in uninfected participants. The risk of subsequent *P. vivax* infections following *P. vivax* in monoinfections was about 12 times increased (RR 12.2, 95% CI 11.0 to 13.6) compared to uninfected participants. When *P. falciparum* parasites were detected in participants with either mono- or mixed-infections during the preceding survey, the risk of subsequent *P. vivax* infection was almost 5 times increased (RR 4.9, 95% CI 4.1 to 5.9) compared to uninfected participants.

**Fig. 1:**
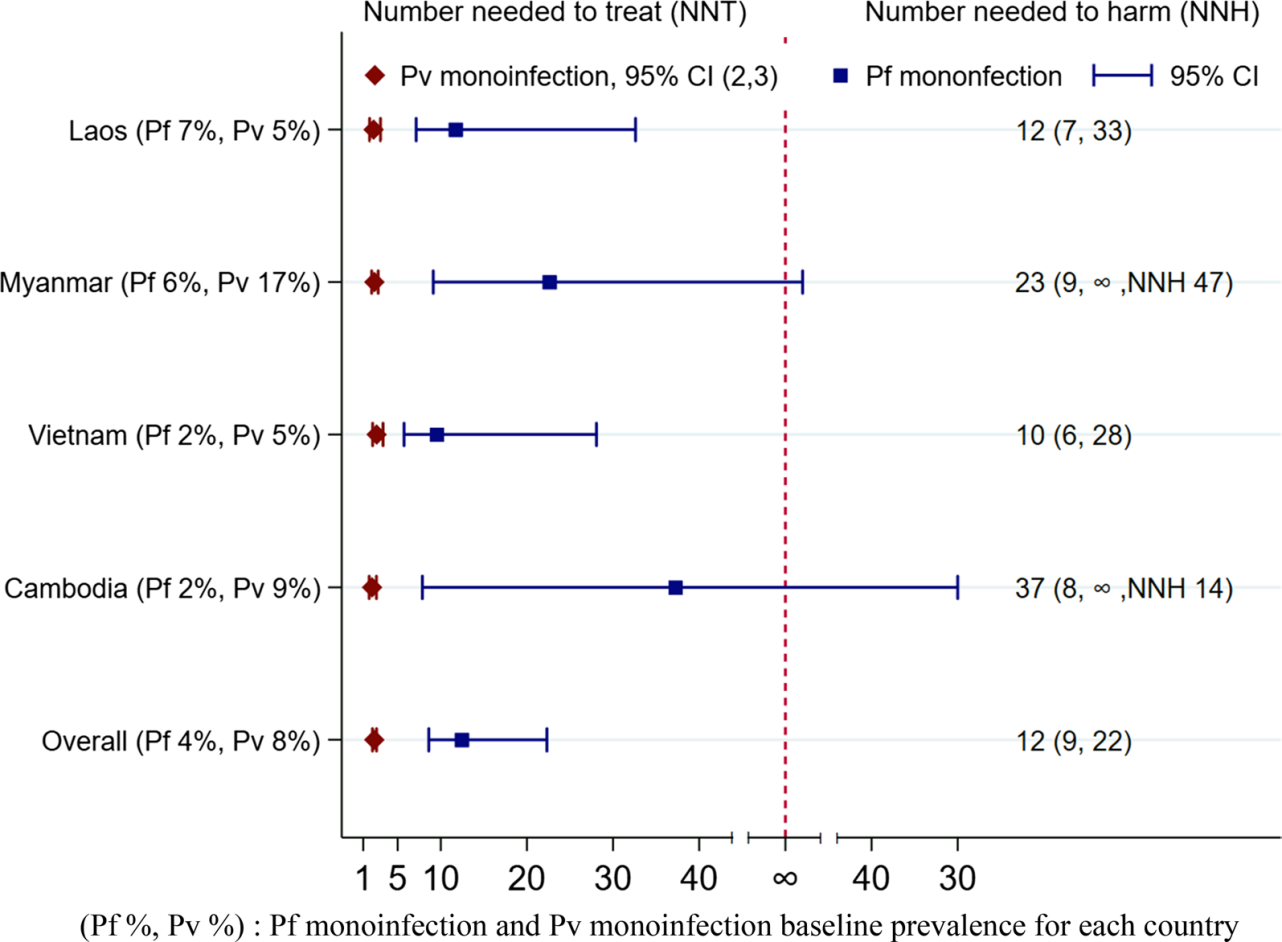
Numbers individuals with *P. falciparum* (blue) or *P. vivax* (red) monoinfections that need to be treated with an appropriate 8-aminoquinoline regimen to prevent one *P. vivax* infection, assuming 99% *P. vivax* radical cure rate by country of the study site. (Pf %, Pv %): Pf monoinfection and Pv monoinfection baseline prevalence for each country

Table 2 summarizes the number of individuals needed to be treated with 8-aminoquinoline in order to prevent one *P. vivax* infection. Assuming that radical cure will prevent 99% of subsequent *P. vivax* infections (relapse), treatment of 12 individuals with asymptomatic *P. falciparum* mono-infections with an appropriate 8-aminoquinoline regimen will prevent one *P. vivax* infection (NNT 12, 95% CI 9 to 22) while treatment of 2 *P. vivax* mono-infected individuals will prevent one sequential *P. vivax* infection (NNT 2, 95% CI 2 to 3). The number of *P. falciparum* mono-infected cases to be treated with 8-aminoquinoline to prevent one *P. vivax* infection varied between study sites (Fig. 1). In Laos, the country with the highest baseline *P. falciparum* prevalence (7%), 12 (95% CI 7 to 33) *P. falciparum* infections would need to be treated with an 8-aminoquinoline to prevent one *P. vivax* infection and in Cambodia, with a baseline *P. falciparum* prevalence of 2%, 37 (95% CI 8 to ∞) *P. falciparum* cases would need to be treated.

**Table 2:** Numbers of individuals needed to be treated with an 8-aminoquinoline to prevent one *P. vivax infection,* assuming 99% *P. vivax* radical cure rate adjusted for correlation among qPCR test result from same individual

Plasmodium parasites detected in the preceding survey	Number of no subsequent *P. vivax* infection, n/N	Probability of no subsequent *P. vivax* infection (95% CI)	Probability no subsequent *P. vivax* with radical cure assumed	RD (95% CI) with 99% *P. vivax* radical cure	NNT (95% CI)
*P. falciparum* (monoinfection)	487/534	0.090 (0.068, 0.119)	0.99	0.080 (0.045, 0.116)	12 (9, 22)
*P. falciparum *+ *P. vivax* (mixed infection)	113/217	0.466 (0.402, 0.540)	0.99	0.456 (0.359, 0.553)	2 (2, 3)
*P. vivax* (monoinfection)	585/1169	0.447 (0.416, 0.480)	0.99	0.437 (0.392, 0.482)	2 (2, 3)
Negative	14,524/15,039	0.036 (0.033, 0.039)	0.99	0.026 (0.022, 0.031)	38 (33, 46)

*RD* risk difference, *NNT* number needed to treat

## Discussion

Following asymptomatic *P. falciparum* monoinfections participants had a 9% probability of having an asymptomatic *P. vivax* infection at the next survey compared to a 4% probability in the absence of previously detected *Plasmodium* species. Only two people with *P. vivax* infections need to be treated to prevent one sequential *P. vivax* infection irrespective in which study site the infections were detected. By contrast, overall 12 asymptomatic monoinfections with *P. falciparum* need to be treated to prevent one subsequent *P. vivax* infection but this number varied by location.

A recent systematic review examined the risk of clinical vivax episodes following clinical falciparum malaria [9]. The investigators thought the risk of clinical vivax malaria episodes following falciparum malaria was mainly determined by the terminal half-life of the antimalarial drug used to treat the falciparum malaria episode and the periodicity of the *P. vivax* relapse pattern. In regions with short relapse periodicity including the GMS the risk was higher than in regions with longer intervals between relapses, i.e. regions further removed from the equator. By day 63 after a presentation with clinical falciparum malaria, independent of the type of schizontocidal drug administered for the falciparum episode, at least 15% of study participants had *P. vivax* parasitaemia in co-endemic countries.

One of the principal differences of the current study to earlier work is the use of asymptomatic infections to estimate probabilities and not clinical malaria episodes. There are good reasons to treat and clear asymptomatic infections in the interest of the infected individual [18] as well as to reduce and ultimately interrupt transmission, but asymptomatic infections may have different epidemiological characteristics and are likely to have different probabilities for subsequent vivax relapse than clinical malaria episodes. Second the current study detected infections in quarterly intervals. Events occurring after *P. falciparum* infection but ending before the next quarterly survey were missed by the current analysis. Using the quarterly surveys and in the absence of appropriate genotyping, we were unable to distinguish vivax re-infections or relapses from persistent infections. A recent analysis of data from the study site in Vietnam showed that asymptomatic *P. vivax* as well as *P. falciparum* infections persisted frequently for months in the absence of a curative treatment [19]. The number needed to treat (NNT) is an epidemiological measure used in communicating the effectiveness of a health-care intervention. The NNTs presented here do not include the reduction in vivax malaria transmission resulting from the implementation of the universal radical cure. The overall benefits of universal radical cure are therefore likely to be even larger than suggested by the NNTs.

## Conclusion

Rational decision making whether to implement universal radical cure should consider benefits relative to safety risks. Considering the tangible and intangible costs of vivax malaria infections and the prospect of interrupting transmission, even treating 37 individuals with falciparum malaria to prevent one *P. vivax* episode, the highest number-needed-to-treat observed, seems justified. However, in the administration of 8-aminoquinoline safety concerns have a high priority. The introduction of robust and accurate tests which allow the quantitative estimation G6PD activity will make the administration of 8-aminoquinoline safer and the licensing of tafenoquine which can be administered as a single dose is likely to increase adherence to the radical cure. Good reasons to implement universal radical cure are accumulating. Whether the potential benefits outweigh the risks remains a judgement call for policymakers and needs to be based on local circumstances specifically the malaria and G6PD deficiency prevalence and the local capacity to diagnose G6PD deficiency correctly.

## Data Availability

The data are available upon request to the Mahidol Oxford Tropical Medicine Research Unit Data Access Committee (http://www.tropmedres.ac/data-sharing) for researchers and following the Mahidol Oxford Tropical Medicine Research Unit data access policy (http://www.tropmedres.ac/_asset/file/datasharing-policy-v1-1.pdf). Queries and applications for datasets should be directed to Rita Chanviriyavuth (rita@tropmedes.ac).

## Abbreviations

°C: degrees Celsius
µL: microlitre
95% CI: 95% confidence interval
DHA: dihydroartemisinin
G6PD: glucose-6-phosphate dehydrogenase
GEE: Generalized Estimating Equation
GMS: Greater Mekong Subregion
M1, M2, M3, …: Month 1, Month 2, Month 3, …
MDA: mass drug administration
mL: mililitre
NNT: number needed to treat
PPQ: piperaquine
RD: risk difference
RR: risk ratio
SLDPQ: single low dose primaquine
uPCR: high-volume ultrasensitive quantitative polymerase chain reaction
Lao PDR: Lao People’s Democratic Republic
MORU: Mahidol-Oxford Research Unit
DP: dihydroartemisinin–piperaquine
Hb: haemoglobin
